# Identifying variation in resistance to the take-all fungus, *Gaeumannomyces graminis* var. *tritici*, between different ancestral and modern wheat species

**DOI:** 10.1186/s12870-014-0212-8

**Published:** 2014-08-02

**Authors:** Vanessa E McMillan, Richard J Gutteridge, Kim E Hammond-Kosack

**Affiliations:** 1 Department of Plant Biology and Crop Science, Rothamsted Research, Harpenden, Herts AL5 2JQ, UK

**Keywords:** Diversity array technology, Disease resistance in wheat roots, Gaeumannomyces graminis, Soil-borne fungal pathogen, Take-all disease, Triticum monococcum

## Abstract

**Background:**

Ancestral wheat relatives are important sources of genetic diversity for the introduction of novel traits for the improvement of modern bread wheat. In this study the aim was to assess the susceptibility of 34 accessions of the diploid wheat *Triticum monococcum* (A genome) to *Gaeumannomyces graminis* var. *tritici (Ggt)*, the causal agent of take-all disease. The second aim was to explore the susceptibility of tetraploid wheat (*T. durum*) and the B genome progenitor species *Aegilops speltoides* to *Ggt*.

**Results:**

Field trials, conducted over 5 years, identified seven *T. monococcum* accessions with a good level of resistance to take-all when exposed to natural inoculum under UK field conditions. All other accessions were highly susceptible or did not exhibit a consistent phenotype across years. DArT marker genotyping revealed that whole genome diversity was not closely related to resistance to take-all within *T. monococcum*, suggesting that multiple genetic sources of resistance may exist within the species. In contrast the tetraploid wheat cultivars and *Ae. speltoides* were all highly susceptible to the disease, including those with known elevated levels of benzoxazinoids.

**Conclusions:**

The diploid wheat species *T. monococcum* may provide a genetic source of resistance to take-all disease that could be utilised to improve the performance of *T. aestivum* in high disease risk situations. This represents an extremely valuable resource to achieve economic and sustainable genetic control of this root disease.

## Background

Bread wheat (*Triticum aestivum*) is the most extensively grown domesticated wheat species and one of the four major food crops of the world. The ascomycete soil-borne fungus, *Gaeumannomyces graminis* var. *tritici* (*Ggt*), causes a devastating root disease of wheat called take-all. Take-all is widespread throughout the major wheat producing areas of the world and the fungus also causes damage to the other cereal species barley, triticale and rye [1]. Take-all is a classic example of a soil-borne pathogen that builds up during consecutive susceptible cereal cropping, greatly reducing the yield and quality of grain obtained. Historically, there is an extensive volume of literature on the search for resistance to take-all disease within elite hexaploid bread wheat cultivars [2],[3]. No wheat cultivars displaying a high degree of resistance to take-all have been described and any smaller differences that have been found are generally considered to be too inconsistent for use in wheat breeding programmes [4],[5]. However, breeding for resistance to take-all remains an important goal as it is environmentally and economical attractive, and would give farmers more freedom in rotational cycles. Genetic resources that have proved valuable for the improvement of wheat have included elite cultivars, landraces and ancestral wild relatives [6].

*Triticum monococcum*, a diploid wheat relative of *T. aestivum*, has been reported to contain many potentially useful traits that could be deployed in the improvement of modern hexaploid wheat, including traits influencing germination under salt and drought stress [7] and resistance to a range of pests and diseases. Examples of the latter include resistance to Russian wheat aphid [8], cereal aphids [9],[10], Hessian fly [11], cereal cyst nematode [12], root lesion nematode [13], eyespot [14], fusarium head blight [15], stem rust [16]-[18], leaf rust [19], powdery mildew [20],[21], septoria leaf blotch [22] and soil-borne cereal mosaic virus [23]. *T. monococcum* (A^m^A^m^) is closely related to the main diploid progenitor of the AA genome of tetraploid durum and hexaploid bread wheat, *T. urartu*[24], but was not itself involved in the hybridisation events that created durum and common bread wheat [25]. *Triticum monococcum* has also not been widely used in wheat breeding so the A^m^ genome represents potentially novel sources of resistance to be exploited in modern wheat improvement [7].

The susceptibility of *Triticum monococcum* to take-all disease has not been widely explored. Mielke [26] reported that some *T. monococcum* lines were slightly less susceptible than other wheat species in greenhouse seedling tests. However when the same lines were tested under field conditions all were very severely infected. Nilsson [27] compiled a summary of the literature on the susceptibility of several hundred grass species to take-all. In this summary there were conflicting results between studies with *T. monococcum* ranging from highly resistant to very susceptible. These differences are potentially due to different accessions being tested between studies.

In this study the main objective was to identify whether a high level of resistance to take-all disease exists within *T. monococcum* by evaluating the susceptibility of 34 *T. monococcum* accessions under field conditions. The 34 *T. monococcum* accessions were chosen to cover a wide range of geographic origins and on the basis of seed availability and good growth under UK field conditions. The accessions were tested in comparison to a number of control species: triticale, rye, oats and hexaploid bread wheat. Generally hexaploid wheat is very susceptible to take-all disease, rye is regarded as moderately to highly resistant and triticale is intermediate in resistance [2],[28]-[30]. Oats is almost completely immune to take-all disease of wheat due to the production of the antifungal compound avenacin in plant root tissues [31]. The whole genome diversity of the *T. monococcum* accessions used in the study was assessed using Diversity Array Technology (DArT). The aim was to identify whether relationships exist between the genetic diversity of the *T. monococcom* accessions and their susceptibility to take-all.

The second main objective was to test the resistance of five tetraploid durum wheat cultivars to take-all disease. The probable ancestor of the progenitor species of the B genome of tetraploid wheat, *Aegilops speltoides*, was also included in one of the field experiments. Two of the tetraploid wheat cultivars, Lahn and Cham 1, are adapted cultivars developed at ICARDA [32]. Cham 1 has been reported to show high yield performance and moderate resistance to drought stress while Lahn exhibits good yield stability under a range of environmental conditions [32],[33]. Two of the other durum wheat cultivars, RWA 9 and RWA 10, also originate from ICARDA and are resistant to the Russian wheat aphid. The final durum wheat cultivar, Alifen, and the diploid goat grass *Ae. speltoides* were included because they are considered to produce different levels of the free benzoxazinoids metabolites 2,4-dihydroxy-7-methoxy-1,4-benzoxazin-3-one (DIMBOA) and 2,4-dihydroxy-1,4-benzoxazin-3-one (DIBOA) [34]. Gordon-Weeks *et al.*[34] reported that both *Ae. speltoides* and Alifen contained higher levels of these metabolites in their root systems than hexaploid wheat or *T. monococcum*. Both of these metabolites have previously been reported in *in vitro* studies to inhibit *Ggt* growth and Wilkes *et al*. [35] suggest that the relative resistance of rye compared to wheat may be the result of the combination of both DIBOA and DIMBOA in rye roots. The aim was to test whether these durum wheat lines of interest and *Ae. speltoides* had an increased level of resistance to take-all disease in the field.

To ensure the robustness of the results obtained and their applicability to modern wheat improvement through plant breeding, all material was tested for resistance to take-all under field conditions in the third wheat position in the rotation. The growing of two successive wheat crops in the previous years before starting the field trials ensured that when environmental conditions were favourable for take-all inoculum build-up over successive seasons there was a reasonably high and uniform disease pressure. For comparison the *T. monococcum* accessions in the 2008–2011 field trials were also evaluated for resistance to take-all disease at the seedling stage under controlled environment conditions in a five week pot test.

Our study reveals a range of susceptibilities to take-all disease within the diploid wheat species *T. monococcum*, including some accessions that consistently displayed high levels of resistance across multiple field trial years. In contrast all of the tetraploid durum wheat cultivars were highly susceptible. We also show that whole genome diversity was not closely related to take-all susceptibility within *T.monococcum*, signifying that multiple genetic sources of resistance may be acting. The seedling pot test was not a reliable indicator of field performance within *T. monococcum*, emphasising the importance of multiple field trials to accurately identify resistant material that has the potential to be exploited in plant breeding programmes. The identification of wheat material with resistance to take-all provides key resources that can now be used for genetic and mechanistic analysis of the wheat – *Ggt* interaction and for use in wheat breeding programmes to improve the performance of modern commercial wheat cultivars against this important root disease.

## Results

### Susceptibility of *T. monococcum* to take-all under field conditions

**Figure 1:**
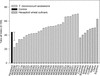
**Intensity of take-all disease for*Triticum*genotypes in the 2006 field trial.** Bar shows the SED for comparison between the genotypes (d.f. = 140, *P* < 0.01).

In the 2005–2006 field season the initial screen of 27 *T. monococcum* accessions revealed a range of susceptibilities to take-all within this diploid wheat species (Figure 1; *P* < 0.01). The mean take-all index was 49.1 with an index of 44.3 for the hexaploid wheat control cv. Hereward, reflecting a moderate to high amount of disease in this year. Under these conditions the majority of accessions had comparable take-all indexes to the hexaploid (*T. aestivum*) wheat cultivars but there was also evidence of potential partial resistance to take-all in some accessions (Figure 1; Take-all index under 30: MDR279 and MDR286).

**Figure 2:**
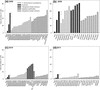
**Intensity of take-all for*Triticum*genotypes in the field trials from harvest years 2008–2011.** In panel **(a)** the bar legend applicable to all four years is given. **(a)** The 2008 field trial. In 2008 there were five replicates per genotype, except for 10 replicates of the *T. monococcum* accessions MDR037, MDR046 and MDR229. Bar shows the SED for comparisons between genotypes sown in 5 replicates (SED min.rep = 9.88, max-min = 8.56, max.rep = 6.99, d.f. = 143, *P* < 0.001). **(b)** The 2009 field trial, **(c)** the 2010 field trial and **(d)** the 2011 field trial. Bars in **(b)**, **(c)** and **(d)** show the SEDs for comparisons between genotypes in those year (2009, d.f. = 84, *P* < 0.001; 2010, d.f. = 124, *P* < 0.001; 2011, d.f. = 104, *P* < 0.05).

Some of the *T. monococcum* accessions were retested in field trials from 2008–2011 and new *T. monococcum* accessions included based on seed availability and results from a limited number of take-all seedling pot tests (RJG, unpublished data). Significant differences in take-all susceptibility between the accessions tested were detected in all four field trials (Figure 2a-d; 2008, 2009 and 2010, *P* < 0.001; 2011, *P* < 0.05). The take-all disease level varied from year to year, with a mean take-all index of 30.3 in 2008 (moderate), 50.9 in 2009 (high) and a mean take-all index of less than 15 in 2010 and 2011 (low). This is most likely a result of differences in environmental conditions between the four growing seasons. The control cereal species, used to benchmark the response of the *T. monococcum* accessions, performed as expected. There were no visible take-all lesions on oats, a non-host to *Ggt*. This agrees with other work done at Rothamsted where oats have been used as a test crop and indicates that the related take-all species *Gaeumannomyces graminis* var. *avenae* is absent from the Rothamsted fields. Rye, as a highly resistant cereal species compared to hexaploid wheat, showed the lowest take-all index out of all the genotypes tested in each of the four field trials. While the wheat x rye hybrid cereal species triticale had an intermediate level of take-all root infection compared to rye and the hexaploid wheat control cultivar Hereward.

Two *T. monococcum* accessions, MDR031 and MDR046, stand out as consistently showing the lowest susceptibility to take-all in the 2008–2011 field trials, intermediate between that of the control species rye and triticale (Figure 2). MDR286, first identified as showing evidence of potential partial resistance to take-all in the 2006 field trial, also shows reasonably low levels of take-all root infection in the 2008, 2010 and 2011 trials. MDR286 was not included in the 2009 trial. Other promising accessions with take-all disease levels similar to triticale include MDR650, MDR232, MDR217 and MDR218. In contrast the *T. monococcum* accessions MDR002, MDR043 and MDR308 were consistently some of the most susceptible to take-all infection, with take-all indexes similar to or above the hexaploid wheat control cv. Hereward. Two accessions, MDR280 and MDR229, performed quite well in 2008 and 2009 when the overall amount of take-all disease was quite high (Hexaploid wheat control cv. Hereward TAI in 2008 = 54.7, 2009 = 59.0). In contrast when there was a lower overall level of disease in 2010 and 2011 (Hexaploid wheat control cv. Hereward TAI in 2010 = 11.0, 2011 = 12.9) these accessions were more susceptible in comparison to the control species and the ranking of the *T. monococcum* accessions in the previous trials.

In each of the four trial years (Figure 2) and the initial screen in 2006 (Figure 1) a number of other hexaploid wheat cultivars were included. In the moderate to high take-all years of 2006, 2008 and 2009 these cultivars displayed relatively high take-all indexes, reflecting the known high susceptibility of modern wheats to take-all. The hexaploid wheat cultivar Solstice (2009, 2010 and 2011) displays a trend towards lower levels of take-all root infection while Robigus (2006 and 2008–2011) was one of the most heavily infected cultivars. Many other hexaploid wheats in the study, such as Cordiale (2006, 2008, 2009 and 2010) and Einstein (2008, 2009 and 2010), did not perform consistently from year to year.

In 2009 and 2010 five tetraploid durum wheat cultivars were evaluated for their susceptibility to take-all (Figure 2b and Figure 2c). In both years all five cultivars showed very high susceptibility to take-all. This is particularly noticeable in 2010, where despite the overall low amount of take-all disease across the trial (mean TAI = 13.7) the five tetraploid cultivars had take-all indexes ranging from 29 to 42. In contrast the hexaploid wheat cultivars (considered to be fully susceptible to take-all) had take-all indexes ranging from only 5.4 to 13.3. In 2010 (Figure 2c), the wild goatgrass *Ae. speltoides* was also included in the field trial. This species exhibited an intermediate level of take-all root infection between the hexaploid and tetraploid wheat cultivars.

### Susceptibility of *T. monococcum* to take-all in a seedling pot test

The seedling pot test revealed a range of susceptibilities to take-all disease for *T. monococcum*, from 13.9% roots infected for MDR217 to 38.1% for MDR280 (Table 1). Rye and triticale were included to compare their known susceptibilities to take-all in the field as adult plants to their performance at the seedling stage. Rye had the lowest level of infection with 2.8% roots infected. Triticale had 11.4% roots infected. By comparison the fully susceptible winter wheat cultivar Hereward had 33.2% roots infected with take-all, revealing that the resistance of rye and triticale to take-all disease is effective at both the seedling stage and as adult plants in the field.

**Table 1:** **Susceptibility of** ***T. monococcum*** **accessions to take-all infection in a seedling pot test**

**Treatment**	**Logit percentage roots with take-all (back transformed means)**
***T.monococcum*** **accessions**	
MDR217	−1.82 (13.9)
MDR031	−1.62 (16.6)
MDR229	−1.42 (19.4)
MDR218	−1.38 (20.0)
MDR026	−1.27 (22.0)
MDR046	−1.12 (24.6)
MDR044	−1.00 (26.9)
MDR650	−0.99 (27.1)
MDR025	−0.95 (27.9)
MDR002	−0.95 (27.9)
MDR286	−0.80 (31.0)
MDR037	−0.80 (31.1)
MDR043	−0.77 (31.6)
MDR308	−0.70 (33.2)
MDR232	−0.67 (33.9)
MDR280	−0.49 (38.1)
Rye	−3.54 (2.8)
Triticale	−2.05 (11.4)
Hereward	−0.70 (33.2)
d.f.	76
SED (logit scale)	0.585
F Probability	<.001

*Triticum monococcum* accessions MDR217, MDR031 and MDR229 were the least infected with take-all in the seedling pot test (less than 20% roots infected) (Table 1). In the field there was also a trend for these varieties to have lower levels of take-all infection. By comparison other partially resistant accessions in the field (MDR046, MDR650, MDR286 and MDR232) did not show any resistance at the seedling stage with the percentage roots infected similar to the highly susceptible accessions from the field (MDR002, MDR043 and MDR308). The results at the seedling stage do not therefore accurately relate to performance under field conditions.

### *T. monococcum* DArT diversity analysis

**Figure 3:**
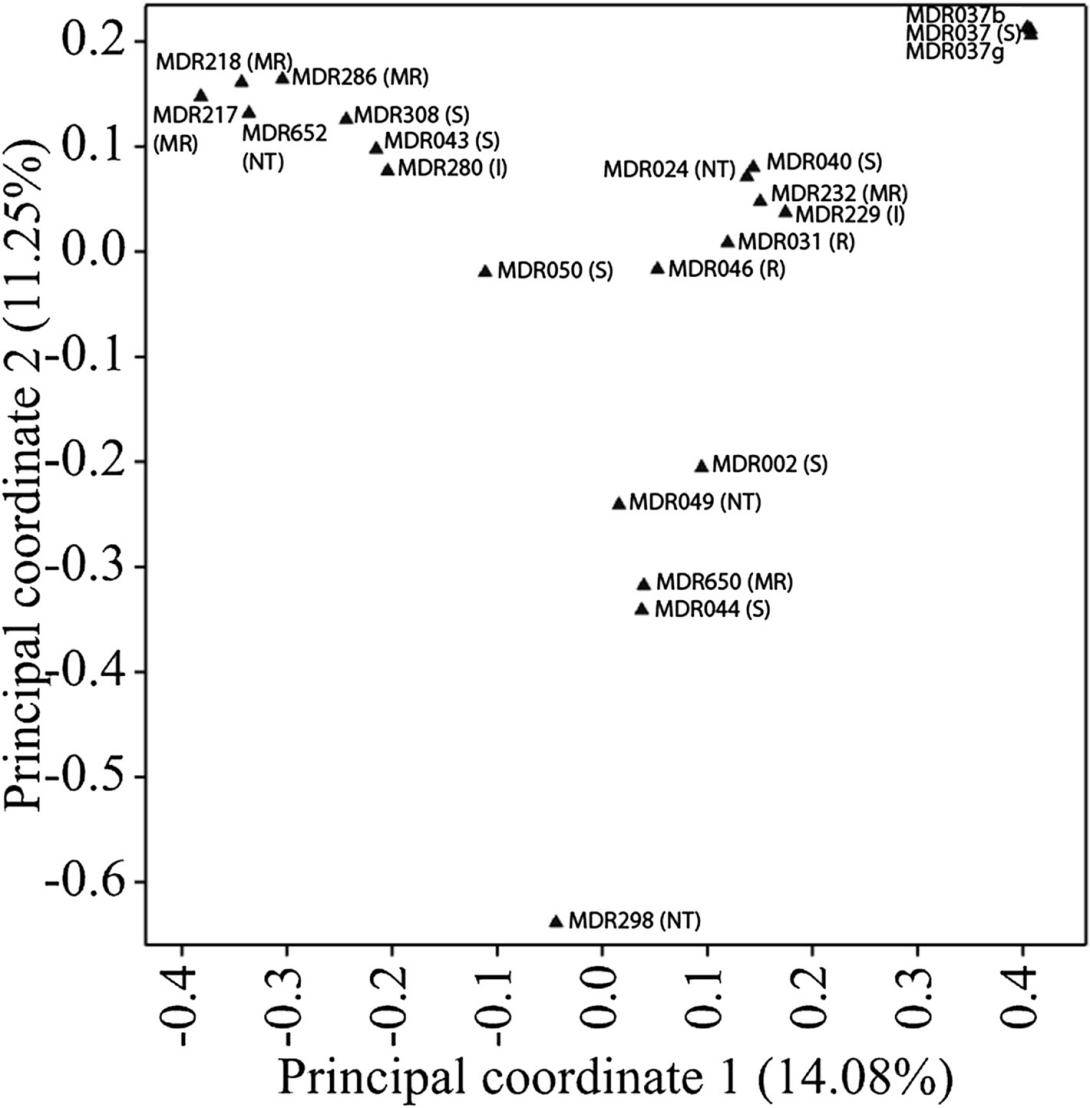
**Principal coordinate analysis of 20** ***T. monococcum*accessions based on 1041 DArT markers.** The diagram shows the position of each accession in the space spanned by the first two coordinates of a relative Jaccard similarity matrix. The accession codes and susceptibility to take-all are inserted in the figure. Susceptibility to take-all is based on the field screening disease index scores reported in this study. Accessions were classified as susceptible (S), moderately resistant (MR), resistant (R), inconsistent performance in different field trials (I), and not tested in the field (NT).

Twenty *T. monococcum* accessions were analysed using diversity arrays technology by Triticarte, Australia (http://www.diversityarrays.com). The accessions were genotyped using over 1000 DArT markers. Polymorphism Information Content (PIC) values ranged from 0.087 to 0.50 with an average PIC value of 0.30. Principal coordinate analysis shows the separation of accessions based on their genotypes (Figure 3). The principal coordinate plot shows the position of each accession in the space spanned by the first two coordinates of a relative Jaccard similarity matrix. These first two coordinates together explained 25.33% of the data variation. There was no strong correlation between this genetic clustering and the susceptibility of the accessions to take-all based on the field trials reported in this study. However, the two accessions most resistant to take-all in the field (MDR031 and MDR046) do cluster quite closely together. Three separate samples of MDR037 were analysed by DArT genotyping using DNA from different seed stocks. These are shown to be grouped very closely together (Figure 3), although there were still some differences between the seed stocks, indicating that the different sources are not genetically pure.

## Discussion

Field experiments conducted in five different growing seasons provide evidence of a reproducible level of resistance to take-all within seven *T. monococcum* accessions. The two most resistant accessions, MDR031 and MDR046, were intermediate in resistance between the species controls rye and triticale. The other five accessions were similar to triticale. No accession was found to contain the almost complete immunity to *Ggt* which is consistently evident in oats. The other 27 *T. monococcum* accessions were highly susceptible to take-all. These experiments provide evidence that a *Triticum* species possesses resistance to the economically important root invading take-all fungus even when tested in high disease pressure situations.

To date, sometimes relatively large and significant differences between hexaploid wheat cultivars have been reported from individual field experiments, but these have generally not been reproducible across sites and seasons [2]. Scott *et al*. [4] suggest that these differences are real but there is a very large influence of the environment on the host-pathogen interaction and resulting susceptibility of wheat cultivars. To identify any differences that would be useful for plant breeding purposes it is therefore very important that accessions are trialled over multiple years and in different fields. In this study we have demonstrated very consistent differences between the susceptibility of *T. monococcum* accessions to take-all in different seasons at both low and high overall natural disease levels under UK field conditions. These results suggest that the most resistant accessions, MDR031 and MDR046, are promising leads to investigate the genetic basis of resistance to take-all and in molecular breeding approaches to improve the performance of *T. aestivum*.

All of the material was tested under field conditions to ensure that any resistance found could have a practical application in wheat breeding programmes for take-all resistance. In glasshouse studies carried out over limited time periods under controlled conditions it is often hard to demonstrate the practical use of any resistance found. At Rothamsted a seedling pot test method was first established to test the pathogenicity of take-all isolates to wheat and rye seedlings [30]. The assay originally used a silver sand-coarse grit mixture in the pots. A modified version of this pathogenicity test using take-all free soil has since been developed at Rothamsted. This protocol uses field soil collected from take-all free fields (fields not sown with cereals) and artificial inoculum addition to assess the infection of seedlings with take-all. Here we evaluated this method as a way of screening the *Triticum monococcum* accessions for resistance to take-all disease. The results obtained in the seedling pot test were found not to accurately reflect the field performance of these accessions, perhaps because the resistance mechanism is not active at the seedling stage. Further modifications are being carried out to the seedling pot test to see if this method can be used as a way to characterise the *Ggt* – *T. monococcum* interaction in more detail.

The genetic relationships between different *T. monococcum* accessions have not been widely explored. Jing *et al*. [36] previously reported on the development of a DArT marker system for *T. monococcum*. The authors found that the clustering of accessions based on their genetic diversity was only moderately associated to their respective countries of origin. There were 13 accessions in common between the DArT genotyping in this study and the study by Jing *et al*. [36]. In addition there were seven accessions (MDR031, MDR049, MDR218, MDR232 MDR280, MDR286 and MDR298) unique to this study and three accessions (MDR001, MDR045 and MDR657) not included from the previous study. The purpose of adding the additional lines was to extend the whole genome diversity analysis to include accessions with moderate to high levels of resistance against take-all disease in the field study and accessions of interest in aphid resistance studies by colleagues at Rothamsted. Despite the differences between the accessions tested in the two studies there was a very similar clustering of accessions and diversity range in both cases. In this study the DArT genotyping revealed that whole genome diversity was not closely related to the susceptibility of *T. monococcum* to take-all. The most resistant accessions, MDR031 and MDR046, were quite closely clustered but other moderately resistant accessions were more diverse. MDR031 and MDR046 were both collected by the Vavilov Institute (St Petersburg, Russia). Their origins are Turkey and Romania, respectively, and they were collected 43 years apart (Table 2) [7]. This suggests that multiple genetic sources of resistance may exist within *T. monococcum* originating from this region of the world. Potentially, this is an advantage from a plant breeding perspective as it could allow different sources to be combined to further improve the level of resistance to take-all.

**Table 2:** ***T. monococcum*** **accessions used in this field study**

**Accession**^**1**^	**Years in the trials**	**Variety**	**Country of origin**	**Year of collection**	**Growth habit**	**Donor centre**^**2**^
MDR001	1	*flavescens*	Algeria		Spring	JIC
MDR002	5	*atriaristatum*	Balkans		Spring	JIC
MDR025	1	*macedonicum*; *pseudoflavescens*	Ukraine	1923	Spring	VIR
MDR026	1	*pseudomacedonicum*	Ukraine	1923	Spring	VIR
MDR031	3	*monococcum*; *macedonicum*	Turkey	1927	Spring	VIR
MDR035	1	*flavescens*; *vulgare*	Austria	1930	Spring	VIR
MDR037	5	*macedonicum*	Armenia	1934	Spring	VIR
MDR040	1	*vulgare; macedonicum*	Bulgaria	1940	Spring	VIR
MDR043	3	*vulgare*	Greece	1950	Spring	VIR
MDR044	2	*hornemannii*	Turkey	1965	Spring	VIR
MDR045	1	*vulgare*	Denmark	1970	Spring	VIR
MDR046	4	*atriaristatum*; *macedonicum*	Romania	1970	Spring	VIR
MDR047	1	*macedonicum*; *vulgare*	Hungary	1970	Winter	VIR
MDR050	1		Italy		Spring	JIC
MDR217	4	1277	Turkey		Spring	USDA
MDR218	4	2592	Turkey		Spring	USDA
*MDR222*	1	3281	Turkey		Spring	USDA
*MDR227*	1	Einkorn	United States		Spring	USDA
*MDR228*	1	2497	Turkey		Spring	USDA
MDR229	4	3962	Spain		Spring	USDA
*MDR232*	3	*nigricultum*	Yugoslavia		Winter	USDA
MDR236	1	I-1-1914	Hungary		Spring	USDA
MDR243	1	2934	Romania		Winter	USDA
*MDR244*	1	K930	Morocco		Spring	USDA
MDR258	1	Einkorn	Israel		Spring	USDA
*MDR261*	1	G2886	Iraq		Spring	USDA
*MDR264*	1	G2900	Turkey		Spring	USDA
*MDR279*	1	G2944	Turkey		Spring	USDA
*MDR280*	4	G2946	Turkey		Spring	USDA
*MDR286*	4	84TK154-034	Turkey		Winter	USDA
MDR303	1	T-1600	Spain		Spring	USDA
*MDR306*	1	957	Former Yugoslavia		Spring	USDA
MDR308	5	DV92	Italy		Spring	UC Davis
MDR650	3	PI355520	Iran			USDA

^1^*T. monococcum* accession information previously published (Jing *et al*. 2007, 2008 and 2009 [7],[22],[36]) Accessions in italics = not previously published.

^2^JIC = John Innes Centre, Norwich, UK; UC Davis = University of California, Davis, CA, USA; USDA = United States Department of Agriculture, Agricultural Research Service, Aberdeen, ID, USA; VIR = N.I. Vavilov Research Institute of Plant Industry, St Petersburg, Russia.

Neither the genetic or mechanistic basis of resistance to take-all observed in some of the *T. monococcum* accessions is known. The diploid *T. monococcum* is relatively closely related to modern tetraploid and hexaploid wheat species, and genetic loci conferring resistance to leaf rust and powdery mildew have already been successfully introgressed into modern wheats [37]-[39]. The smaller diploid genome of *T. monococcum* and the contrasting susceptibilities of accessions to take-all make this species ideal for genetic studies of resistance. Already several mapping populations are being developed within the Wheat Genetic Improvement Network programme for this purpose (http://www.WGIN.org.uk). However, these mapping populations once generated will then need to be evaluated over several field seasons to ensure that the resistance identified in this study, which is effective in reducing disease levels in the root system until crop harvest, is correctly mapped. Such genetic analysis should reveal whether the trait is controlled by a single locus or multiple loci and whether there are distinct genetic sources of resistance in different accessions. Introgression of *T. monococcum* into modern hexaploid bread wheat is also currently underway using Paragon lines harbouring the homoeologous pairing locus mutation *ph-1*[40]. Some of the resulting F_1_ lines will be field tested in a 3^rd^ wheat situation alongside the two parental lines to give an early indication of the take-all resistance phenotype in a 50% *T. aestivum* background.

In the case of rye (*Secale cereale*) there are numerous studies, in different regions of the world, reporting on the good level of resistance to take-all disease within this species [27],[28],[30],[41],[42]. However, so far it has not been possible to identify the genetic basis of resistance and subsequently introgress this resistance into hexaploid wheat. The introduction of single rye chromosomes into wheat chromosome addition lines did not transfer resistance from rye to wheat, signifying that resistance is likely to be polygenic and involve loci on multiple chromosomes [28]. Genetic analysis of the resistance trait in rye is also made much more difficult by the lack of variation between rye cultivars in their resistance to take-all. In contrast within *T. monococcum* we have had the opportunity to develop mapping populations between contrasting accessions that can now be used to investigating the genetic basis of the resistance phenotype under field conditions. The potential success of introgressing resistance from *T. monococcum* into hexaploid wheat will depend on the effect and number of loci that are identified. So far these *T. monococcum* accessions have also only been tested under local UK field conditions. It would be interesting to assess their performance in other parts of the world with their different *Ggt* pathogen populations, climatic conditions and crop husbandry systems to assess the usefulness of this species to improve resistance to take-all disease on a wider scale.

The main focus of this study was to explore the resistance of *Triticum monococcum* roots to take-all disease. In addition a number of other hexaploid and tetraploid wheat cultivars were included for interest and inter-comparison. There was a trend for some relatively consistent differences between the hexaploid wheat cultivars tested. Hereward is consistently very susceptible to take-all disease and was included in the field trials as a cereal species control for full susceptibility. Hereward is a commercial elite cultivar, released in 1991, with an important position in UK wheat farming and bread making. The reliable quality of the grain meant that Hereward became a preferred choice for farmers growing milling wheat and it is still grown on small areas today. Other hexaploid wheat cultivars in this study also performed fairly consistently; for example Solstice was one of the least susceptible cultivars and Robigus was usually more heavily infected. This data does suggest that there are real differences between modern wheat cultivars in their susceptibility to disease. However, most of the differences between the hexaploid wheats were relatively small compared to the larger range of susceptibilities within *T. monococcum*.

There have been extensive searches for resistance to take-all within bread wheat but there is much less available information on the susceptibility of durum wheat to take-all. In the work presented here the five durum wheat cultivars, tested in 2009 and 2010, were all very susceptible to take-all disease. *Aegilops speltoides* (a probable ancestor of the progenitor species of the B genome of tetraploid wheat), was also included in the 2010 field trial and found to be susceptible to take-all. The Chilean durum wheat cultivar Alifen and the diploid goat grass *Ae. speltoides* have previously been reported as producing higher levels of the benzoxazinoids DIMBOA and DIBOA in their roots than hexaploid wheat [34]. These metabolites have been implicated in resistance to a range of pests and pathogens including insects, fungi, nematodes and weeds [43]. DIMBOA and DIBOA have also both been reported to inhibit the growth of the take-all fungus in *in vitro* growth tests [35]. However, our study shows no evidence of any resistance against the take-all fungus for either the durum wheat cultivar Alifen or *Ae. speltoides*. Therefore, it is unlikely that these secondary metabolites are able to provide prolonged protection against take-all disease in the field. Even under the overall low disease situation in 2010 both of these cultivars were extremely susceptible to take-all, even more so than the hexaploid wheat cultivars which are considered to be fully susceptible. This provides evidence that the B genome lineage is perhaps unlikely to be a useful source of resistance to the take-all fungus. However, the higher susceptibility of tetraploid wheat compared to hexaploid wheat in this study could suggest that the introduction of the D genome into modern hexaploid wheat has increased the resistance of wheat to take-all.

## Conclusions

Robust field protocols for effectively assessing the susceptibility of cereal germplasm to take-all disease have been developed. Resistance to the root disease, effective over different field sites and seasons, was identified within the diploid wheat species *Triticum monococcum*. This reliable root resistance to the disease within a *Triticum* species represents a key step towards the potential genetic control of the disease. In contrast the tetraploid durum wheat cultivars were all highly susceptible to take-all, including those which are known to produce elevated levels of benzoxazinoids. The results confirm that ancestral wheat relatives are vital resources for the improvement of modern hexaploid bread wheat against biotic stresses.

## Methods

### Plant material

The 34 *T. monococcum* accessions used in this field study had originally been collected from 19 countries (Table 2). The further details for 23 of these accessions were first published in previous studies (Jing *et al*. [7],[22],[36]). Thirty hexaploid (AABBDD) wheat cultivars (Table 3) and the five tetraploid (AABB) wheat cultivars (Lahn, Cham 1, RWA 9, RWA 10 and Alifen) were also included in the field study. Control cereal species for comparison included oats (cv. Gerald), rye (cv. Carotop), triticale (cv. Trilogie) and hexaploid bread wheat (cv. Hereward).

**Table 3:** Hexaploid wheat cultivars used in this field study

**Cultivar**	**Years in the trials**	**Year first listed**^**1**^	**Growth habit**
Alchemy	2	2006	Winter
Bantam	2	NR (2008)	Winter
Battalion	1	2007	Winter
Bobwhite^2^	2	-	Spring
Cassius	2	2009	Winter
Chinese Spring^3^	1	-	Spring
Claire	1	1999	Winter
Consort	1	1995	Winter
Cordiale	4	2004	Winter
Duxford	3	2008	Winter
Einstein	3	2003	Winter
Equinox	1	1997	Winter
Gallant	1	2009	Winter
Hereford	1	NR (2007)	Winter
Hereward	5	1991	Winter
Hyperion	1	2006	Winter
Invicta	1	2010	Winter
Istabraq	3	2004	Winter
JB Diego	1	2008	Winter
Lear	2	NR (2008)	Winter
Napier	1	2000	Winter
Panorama	1	2009	Winter
Paragon	2	1999	Spring
Q Plus	1	2009	Winter
Robigus	5	2003	Winter
Shogun	1	NR (2008)	Winter
Solstice	3	2002	Winter
Tybalt	1	2003	Spring
Welford	1	2004	Winter
Xi19	2	2002	Winter
Zebedee	1	2007	Winter

^1^ Date first listed in the UK Recommended List (RL). NR = not recommended (first candidate year).

^2^ Bobwhite was developed in the 1970s at CIMMYT.

^3^ Chinese Spring is a Chinese Land Race.

### Field trials

Five field trials, one in each of the harvest years in 2006 and 2008–2011, were set up to evaluate the susceptibility of *T. monococcum* to take-all disease (Table 4). All of the trials were sown in the autumn on the Rothamsted farm (Hertfordshire, UK) as third wheat crops in the rotation for an expected high natural take-all disease pressure. Trials were set up as randomised block designs of five replicates of each *T. monococcum* accession, except that in 2008 there were two plots per block of three of the accessions (MDR037, MDR046 & MDR229). Plots measured 50 cm by 50 cm and 50-cm paths of bare soil were used to separate plots. Each 3 row plot was hand sown with 60 seeds per plot.

**Table 4:** **Field experiments used to assess the resistance of** ***T. monococcum*** **and tetraploid wheat to take-all**

**Harvest year (field trial code)**	**Rothamsted field**	**Sowing date**	**Treatments**^**1**^	**Date sampled**	**Growth stage (GS)**
2006 (06/R/WW/615)	Delafield	06/10/05	27 *T. monococcum* accessions, 1 control cereal species, 8 hexaploid wheat cultivars	07/07/06	71-73
2008 (08/R/WW/810)	Long Hoos I&II	19/10/07	16 *T. monococcum* accessions, 4 control cereal species, 13 hexaploid wheat cultivars	01/07/08	71-73
2009 (09/R/WW/911)	Stackyard	20/10/08	5 *T. monococcum* accessions, 5 tetraploid wheat cultivars, 3 control cereal species, 9 hexaploid wheat cultivars	09/07/09	71-73
2010 (10/R/WW/1034)	West Barnfield	28/10/09	13 *T. monococcum* accessions, 5 tetraploid wheat cultivars, 3 control cereal species, 10 hexaploid wheat cultivars, 1 *Aegilops speltoides* accession	01/07/10	73
2011 (11/R/WW/1109)	Claycroft	29/10/10	12 *T. monococcum* accessions, 3 control cereal species, 12 hexaploid wheat cultivars	07/07/11	71-73

^1^Control cereal species = hexaploid wheat cv. Hereward in 2006; oats, rye, triticale and hexaploid wheat cv. Hereward in 2008; rye, triticale and hexaploid wheat cv. Hereward in 2009, 2010 and 2011.

Over these five years of trials, 34 *T. monococcum* accessions were evaluated (Table 2). In the 2006 field trial 27 accessions were chosen for an initial screening. In 2008–2011 the *T. monococcum* accessions were selected based on extra information on their phenotypic and genetic diversity in other studies [7],[22],[36], the results of the previous field trials and a limited number of take-all wheat seedling pot tests with some of the accessions (RJG, unpublished data). Fertiliser was applied to the trials according to the standard practice of the Rothamsted farm. No plant growth regulator or fungicides were applied so that the susceptibility of the *T. monococcum* accessions to foliar and stem base diseases could be recorded if appropriate. The foliar and stem base disease data are not reported in this study. In 2009 one dose each of the fungicides Unix® and Allure® were applied in error. Neither of these fungicides has any reported activity against *Ggt* so the trial was not compromised in terms of the take-all study. *Triticum monococcum* is very sensitive to herbicide application. Therefore, a maximum of one dose of the herbicide Pacifica® was applied in the spring where required. In 2008 one dose each of the herbicides Arelon® 500 and Stomp® 400 SC were applied in error in the autumn. However the *T. monococcum* plots did not seem adversely affected by this one dose and showed good establishment in the spring.

### Crop sampling and disease assessment

Plant samples (3 × 20 cm lengths of row per plot) were taken from each field trial at the beginning of July (Growth stage 71–73, milk development). Plant samples were transported back to the field laboratory, roots washed free from soil, the tops chopped off and the remaining stem bases and root systems air dried in a polytunnel for 4–5 days and then stored at room temperature before assessment for take-all disease. Stored dried whole plant root systems were soaked in water for approx. 15–20 minutes and then assessed in a white dish under water and scored for take-all to calculate a take-all index (TAI) [44]. The proportion of roots infected for each whole plant root system was estimated and graded into six categories: no symptoms, slight 1 (1-10% roots infected), slight 2 (11-25%), moderate 1 (26-50%), moderate 2 (51-75%) and severe (more than 75%). From this a take-all index was calculated for each plot: (1 × percentage plants in slight 1 category) + (2 × percentage plants in slight 2 category) + (3 × percentage plants in moderate 1 category) + (4 × percentage plants in moderate 2 category) + (5 × percentage plants in severe category); divided by the number of categories slight 1 to severe (5); maximum TAI 100. Comparisons were made using the analysis of variance procedure in Genstat (VSNI, Hemel Hempstead, UK) [45]. Significant effects were supposed when p ≤ 0.05.

### Seedling pot test

A seedling pot test on the 16 *T. monococcum* accessions from the 2008–2011 field trials was set up to evaluate their susceptibility to take-all under controlled environment conditions. Similar to the field trials, the control species rye (highly resistant under field conditions), triticale (intermediate resistance) and the winter wheat cultivar Hereward (fully susceptible) were also included in the pot test. This 5 week seedling pot test protocol used field soil collected from take-all free fields (fields not sown with cereals) and artificial *Ggt* inoculum addition to assess the infection of seedlings.

Soil was collected in summer 2009 from fallow areas in the Rothamsted field ‘Great Field IV’. Large stones were removed and the soil was crumbled and stored in buckets at room temperature. Buckets of soil were mixed together before use in the pot test. Sand-maize meal inoculum of the take-all fungus was prepared by first filling 500 ml conical flasks with 100 g horticultural grade silver sand, 3 g maize meal and 10 ml of distilled water. Flasks were autoclaved twice, with 48 hours between autoclaving. A mixture of 14 *Ggt* isolates were used in the test as representative of a field population with two flasks per individual isolate prepared. The flasks were inoculated with agar discs (6-mm diameter, cut with a cork borer) from fungal cultures on potato dextrose agar, adding three discs per flask and using one isolate per flask. The sand-maize meal cultures were then incubated at room temperature for 6 weeks, with shaking once a week for even colonisation. Sand-maize meal inoculum of different isolates was then mixed together in sterilised 1000 ml conical flasks.

A mixture of 150 g take-all free soil, 100 g damp coarse sand and 50 g of a 1:50 dilution of sand-maize meal *Ggt* inoculum in silver sand (mixed in a plastic bag) was used in the pot test. This mixture was transferred into an 11-cm-tall plastic cup which contained a basal layer of 50 cm^3^ damp sand over four 3-mm-diameter drainage holes in the cup. Ten seeds from a single accession were then placed on the soil surface and covered with a thin layer of horticultural grit. Five replicates were set up per treatment. A control treatment without addition of *Ggt* sand-maize meal was set up with the winter wheat cultivar Hereward to ensure the soil used was free from take-all. All pots were then gently watered and placed in a controlled environment room in a randomised design (16 hour day, day/night temperatures 15/10°C, twice weekly watering). After 5 weeks the plants were removed and their roots washed out with water before disease assessment. The total number of roots and then the number of roots infected with take-all were recorded so that the percentage of roots infected could be calculated. Comparisons were made using the analysis of variance procedure in Genstat (VSNI, Hemel Hempstead, UK) [45]. Significant effects were supposed when p ≤ 0.05.

### DArT diversity analysis

DArT marker assays were carried out by Triticarte, Australia (http://www.diversityarrays.com). Twenty *T. monococcum* accessions were genotyped in the array (Jing *et al*. [36]) using 1041 markers. Sixteen of the accessions were chosen from the take-all field trial study to include the highly and moderately resistant accessions and also a range of the susceptible accessions. The other 4 accessions were included in the array because these were of interest in relation to other traits studied by colleagues at Rothamsted including aphid resistance and Septoria leaf blotch resistance. Colleagues at Rothamsted had obtained contrasting results in aphid feeding tests with different sources of MDR037 seed (L. Smart, unpublished). Three samples of MDR037, originating from different seed stocks, were therefore analysed. Accessions were scored at each marker for the presence or absence of the DNA fragment of interest, represented by a ‘1’ or ‘0’. If a marker could not be reliably scored for a particular sample this was treated as missing data and scored as ‘-’. A Jaccard similarity matrix was generated and used to carry out a principal coordinate analysis (PCoA) in Genstat (VSNI, Hemel Hempstead, UK) [45].

The relationship between resistance to take-all and genotype for the *T. monococcum* accessions was explored by inspecting the genetic clustering of the accessions in the PCoA plot in comparison to their take-all levels in the third wheat field trials, with particular focus on 2008 and 2009 when there was better discrimination between accessions under the higher overall disease levels. The *T. monococcum* accessions were classified as susceptible (S), moderately resistant (MR), resistant (R), inconsistent (I), and not tested for take-all resistance (NT). Susceptible accessions generally had a take-all index above triticale and also sometimes the hexaploid wheat control cv. Hereward. Moderately resistant cultivars had a take-all index below cv. Hereward and were generally close to triticale. Resistant accessions were designated as those that had a take-all index intermediate between rye and triticale. Inconsistent (I) accessions were those that gave contrasting results in different field trials years.

## Competing interests

The authors declare that they have no competing interests.

## Authors’ contributions

RG and KHK conceived the study and provided overall supervision of the research. VM and RG carried out the experimental work. All authors critically examined and analysed the datasets. VM and KHK wrote the manuscript. All authors read and approved the final manuscript.
